# A genome-wide association study in a large F2-cross of laying hens reveals novel genomic regions associated with feather pecking and aggressive pecking behavior

**DOI:** 10.1186/s12711-017-0287-4

**Published:** 2017-02-03

**Authors:** Vanessa Lutz, Patrick Stratz, Siegfried Preuß, Jens Tetens, Michael A. Grashorn, Werner Bessei, Jörn Bennewitz

**Affiliations:** 1 Institute of Animal Science, University of Hohenheim, 70599 Stuttgart, Germany; 2 Division of Functional Breeding, Department of Animal Sciences, Georg-August-University Göttingen, 37077 Göttingen, Germany

## Abstract

**Background:**

Feather pecking and aggressive pecking in laying hens are serious economic and welfare issues. In spite of extensive research on feather pecking during the last decades, the motivation for this behavior is still not clear. A small to moderate heritability has frequently been reported for these traits. Recently, we identified several single-nucleotide polymorphisms (SNPs) associated with feather pecking by mapping selection signatures in two divergent feather pecking lines. Here, we performed a genome-wide association analysis (GWAS) for feather pecking and aggressive pecking behavior, then combined the results with those from the recent selection signature experiment, and linked them to those obtained from a differential gene expression study.

**Methods:**

A large F2 cross of 960 F2 hens was generated using the divergent lines as founders. Hens were phenotyped for feather pecks delivered (FPD), aggressive pecks delivered (APD), and aggressive pecks received (APR). Individuals were genotyped with the Illumina 60K chicken Infinium iSelect chip. After data filtering, 29,376 SNPs remained for analyses. Single-marker GWAS was performed using a Poisson model. The results were combined with those from the selection signature experiment using Fisher’s combined probability test.

**Results:**

Numerous significant SNPs were identified for all traits but with low false discovery rates. Nearly all significant SNPs were located in clusters that spanned a maximum of 3 Mb and included at least two significant SNPs. For FPD, four clusters were identified, which increased to 13 based on the meta-analysis (FPD_meta_). Seven clusters were identified for APD and three for APR. Eight genes (of the 750 investigated genes located in the FPD_meta_ clusters) were significantly differentially-expressed in the brain of hens from both lines. One gene, *SLC12A9*, and the positional candidate gene for APD, *GNG2*, may be linked to the monomanine signaling pathway, which is involved in feather pecking and aggressive behavior.

**Conclusions:**

Combining the results from the GWAS with those of the selection signature experiment substantially increased the statistical power. The behavioral traits were controlled by many genes with small effects and no single SNP had effects large enough to justify its use in marker-assisted selection.

**Electronic supplementary material:**

The online version of this article (doi:10.1186/s12711-017-0287-4) contains supplementary material, which is available to authorized users.

## Background

Feather pecking in laying hens is a serious economic and welfare issue that can be observed in commercial and non-commercial chicken flocks. In spite of extensive research on feather pecking during the last decades, the motivation for this behavior is still unclear. The most widespread theory on the origin of feather pecking is that it is a redirected feeding and foraging behavior [1]. Some authors reported that feather pecking is related to dust-bathing [2]. Environmental factors such as light intensity [3], stocking density [4], and food form [5] can influence feather pecking. Feather pecking behavior has also been associated with fear [6–9]. Other studies suggested that the underlying motivation for feather pecking is feather eating [10–13] or that it is the consequence of a general hyperactivity disorder [14]. Feather pecking is often confounded with aggressive pecking but these two behaviors are clearly distinguishable, both in terms of form and motivation; aggressive pecks are delivered in an upright body posture, are mainly directed to the head of the other birds and aim at establishing and maintaining social hierarchy [15], while feather pecking is performed in a non-aggressive posture. Reports on the relationship between aggressive pecking and feather pecking show no consistent trend. While some authors found no correlation between the two behaviors, positive genetic and phenotypic correlations have been reported in lines selected for high and low feather pecking and their F2-crosses [16, 17]. Depending on the definition of the trait, study design, age of hens, statistical model applied, and data collection period, heritability estimates for feather pecking are low to moderate and range from 0.1 to 0.4, while heritability estimates for aggressive pecking range from 0.04 and 0.14 [17–20].

In a previous study, we analyzed two divergent lines that were selected for 11 generations for high (HFP) and low (LFP) feather pecking, respectively [20, 21]. We estimated genetic parameters within the lines and the phenotypic trend across generations. From the first round of selection onwards, the two lines differed in their means for feather pecking bouts. The highest selection response on the phenotypic scale was obtained during the first rounds of selection and thereafter, no clear trend was observed in the HFP line. The LFP line showed a constant low level of pecking behavior across the 11 generations of selection. Heritabilities of feather pecking estimated based on linear mixed models were equal to 0.15 and 0.01 in the HFP and LFP lines, respectively. The distribution of feather pecking bouts within each line and for each round of selection are discussed in detail in [21].

In addition, we performed a genome scan to map selection signatures in these two divergent HFP and LFP lines using an F_ST_-based approach [20]. The analysis provided 17 genome-wide significant single-nucleotide polymorphisms (SNPs), most of which were located in clusters, which supports the presence of selection signatures.

These HFP and LFP lines formed the base population of the F2-population used in the current study, in which a genome-wide association analysis (GWAS) for feather pecking and aggressive pecking behavior was performed. The results obtained were combined with those from the previous selection experiment [20] in a meta-analysis, and then linked to those obtained from a differential gene expression study.

## Methods

### Experimental population

Chickens from a White Leghorn line were divergently selected for low and high feather pecking for 11 generations, resulting in a LFP and a HFP line. Selection took place for five generations at the Danish Institute of Animal Science [18] and then for five additional generations at the Institute of Animal Science, University Hohenheim, Germany [20]. From these two lines, a large F2 cross was established. Five sires and ten dams from generation 11 of each line were used to generate 10 F_1_ families. Each HFP sire was crossed with two LFP full-sib dams and vice versa. Then, 10 F_1_ sires were used to generate the F_2_ families. Each sire was mated with eight F_1_ hens four times by artificial insemination. A total of 960 F2 offspring were produced in four hatches, with an interval of 3 weeks between hatches.

### Phenotypes

At 27 weeks of age, feather pecks delivered (FPD) and aggressive behavior [aggressive pecks delivered (APD) and aggressive pecks received (APR)] were recorded in groups of 36 to 42 hens. The applied ethogram was according to Savory [22] and Bessei et al. [16] and was as follows. Feather pecking was defined as a non-aggressive behavior and included forceful pecks, sometimes with feathers being pulled out and the recipient hen either tolerating this action or moving away. Aggressive pecking was defined as fast pecks towards the head and body of conspecifics. Usually, the hen that was attacked moved away but may have incurred tissue damage. For the behavioral observations, the hens were marked with numbered plastic batches on their backs. Seven observers visually recorded feather pecking and aggressive pecking within each pen during 20-min sessions for three consecutive days during daytime. Hatches 3 and 4 were observed twice for three consecutive days. The total number of observers varied between five and seven persons per observation day. The numbers of FPD, APD, and APR were summed over the entire observation period and standardized to an observation period of 420 min. Heritabilities of FPD (APD, and APR), estimated with a linear mixed model in this F2 cross, were equal to 0.12 (0.27, and 0.27) [23]. Genetic and phenotypic correlations of 0.2 and 0.09, respectively, were obtained between FPD and APD [9]. Correlations of estimated breeding values between FPD and APR and between APD and APR were 0.18 and −0.23, respectively [17].

### Genotypes

A total of 817 F2 hens were genotyped with the Illumina 60K chicken Infinium iSelect chip. For the remaining hens no samples were collected. A total of 57,636 SNPs were genotyped and after data filtering, 29,376 SNPs remained in the dataset. Based on positional information according to the chicken genome assembly galGal2.1, SNPs that were located on the sex chromosomes W or Z or in the linkage groups LGE22C19W28_E50C23 or LGE64, and SNPs that were not allocated to a specific chromosome or linkage group were excluded. In addition, SNPs with minor allele frequencies (MAF) lower than 0.03 and SNPs with a call frequency lower than 0.95 were filtered out.

### Statistical analysis

In order to investigate the mapping resolution of the design, the linkage disequilibrium (LD) structure was investigated for the first nine chromosomes i.e. GGA1 to GGA9 (GGA for *Gallus gallus* chromosome). The Beagle Genetic Software Analysis [24, 25], which is included in the synbreed R package [26], was used to phase haplotypes and then the common LD measure r^2^ was estimated using PLINK [27] for pairs of SNPs that were <5 Mb apart across the autosomes.

GWAS are frequently conducted using mixed linear models (e.g., [28]). In its simplest form, such models include a general mean, a fixed SNP effect and a random family effect. The latter is important to capture population stratification effects and, hence, to prevent inflation of type I errors (e.g., [29]). Previous studies showed that FPD, APD and APR are not normally distributed and that Poisson models should be used for the statistical analyses [17, 20]. Poisson models with fixed and random effects belong to a class of generalized linear mixed models (GLMM). Due to the lack of a closed form of expression of the likelihood for these models, approximate likelihood techniques are often used, as for example in the software ASReml [30]. However, for hypothesis testing, the behavior of these techniques has not been sufficiently well investigated, and Collins [31] recommended that GLMM should not be used for this purpose. Therefore, we used the following generalized linear model based on the Poisson distribution and no random effects for single-marker association analysis:

1 $$\eta_{ijm} = H_{j} + S_{i} + D_{i} + b_{m} x_{im} ,$$

where *η*_*ijm*_ is the linear predictor for hen *i* and SNP *m*, *H*_*j*_ is the fixed hatch effect, *S*_*i*_ and *D*_*i*_ are the fixed sire and dam effects, respectively, *x*_*im*_ denotes the number of copies of the minor allele of SNP *m* (*x* = 0, 1, or 2), and *b*_*m*_ is the regression coefficient for SNP *m*. Thus, instead of fitting a random family effect, we included fixed sire and dam effects in the model to account for population stratification effects.

In a previous study, we detected substantial permanent environmental effects for FPD, APD and APR [17], which could also be caused by dominant gene effects. Because dominance and additive gene effects tend to be correlated such that larger dominance deviations are observed for genes with larger additive effects [32], we tested only genome-wide significant SNPs from Model (1) or from the meta-analysis (described below) for dominance effects using the following Poisson model:

2 $$\eta_{ijm} = H_{j} + S_{i} + D_{i} + b_{m} x_{im } + \tilde{b}_{m} z_{im}$$

where *z*_*im*_ is an indicator variable, which is 1(0) if the individual is heterozygous (homozygous) at SNP *m* and $\tilde{b}_{m}$ is a fixed regression coefficient, which is a dominance estimate. The other terms are defined as in Model (1).

To correct for multiple-testing, we applied a Bonferroni-type correction as:

$$p_{genome - wide} = 1 - \left( {1 - p} \right)^{{\# {\text{SNP}}}} ,$$

where the number (#) of SNPs was equal to 29,376. The genome-wide significance level was set at *p*_*genome*-*wide*_ ≤ 0.05. Because Bonferroni’s correction is very conservative, we considered an additional nominal significant level; i.e. *p* < 5 × 10^−5^. To estimate the number of false positives among the significant SNPs, we calculated a false discovery rate (FDR) *q* value for each association test by using the software QVALUE [33]. The FDR *q* value of the significant SNP with the largest *p* value provided an estimate of the proportion of false positives among the significant SNPs.

A meta-analysis was performed using the data from the selection experiment and the F2-cross experiment. We combined the *p* values from both studies using the inverse Chi square method of Fisher [34], known as Fisher’s combined probability test, as follows:

$$\chi_{2k}^{2} \sim - 2\mathop \sum \limits_{i = 1}^{k} \ln \left( {p_{i} } \right),$$

where *p*_*i*_ is the *p* value for the *i*th hypothesis test and *k* is the number of studies being combined (i.e., *k* = 2 in our study). The significance levels were used for the *p* value obtained from the meta-analysis were the same as those for the GWAS (Model 1).

### Cluster identification

We assumed that a causative mutation is in LD with several SNPs, and thus built clusters of SNPs, which provided strong evidence for trait-associated chromosomal regions compared to single significant SNPs, although of course it cannot be guaranteed that the mutation is within these clusters. A cluster contained at least two significant SNPs (≤5 × 10^−5^), with a maximum distance of 3 Mb between them. The bounds of each cluster were identified using the LD structure as well as the *p* values of SNPs with lower statistical support, as follows. Starting from the midpoint of the cluster of significant SNPs (*p* ≤ 5 × 10^−5^) and moving in both directions up to 1.5 Mb on each side, we searched for weakly significant SNPs. The weakly significant SNPs (*p* ≤ 5 × 10^−4^) at a maximum distance of 1.5 Mb from the cluster midpoint in both directions were used as the cluster bounds.

### Differential gene expression analysis

Within each FDP_meta_ cluster, genes were investigated for differential expression. Expression data were generated in an earlier study [35]. In brief, the brains of nine hens each from the HFP and LFP line were collected after slaughter. RNA was extracted from the whole brain, reverse-transcribed into cDNA and then converted into labeled cRNA by *in vitro* transcription. Following this procedure, 1.65 µg of each single cRNA sample was hybridized on the Chicken Gene Expression Microarray (4 × 44 K format, Agilent Technologies) and fluorescent signal intensities were detected. The quantile-normalized and log2-transformed data were averaged across the hens within each line. A total of 1083 transcripts included in the microarray gene expression chip were located within the FDP_meta_ clusters. The average expression levels of these genes only were compared between the two lines using a standard Welch *t* test. Correction for multiple-testing was performed using Bonferroni’s test, assuming 1083 independent tests. Sequences of probes with no assigned gene or only a LOC number were subjected to BLAST analysis against the most recent genome database galGal 5.0 (assembly GCA_000002315.3) to identify the corresponding gene. Results of the expression analysis were subsequently compared to the candidate genes that were identified within the associated clusters. Clusters that contained differentially-expressed transcripts were checked for potential enrichment of those transcripts, because this indicates the presence of cis-acting QTL. The corrected *p* values obtained in the original study [35] were used to separate transcripts into three categories of significance i.e. *p* ≤ 0.1, *p* ≤ 0.05, and *p* ≤ 0.01, respectively. For each of these categories, the proportions of significantly differentially-expressed genes within clusters were compared to genome- and chromosome-wide proportions.

## Results

**Fig. 1:**
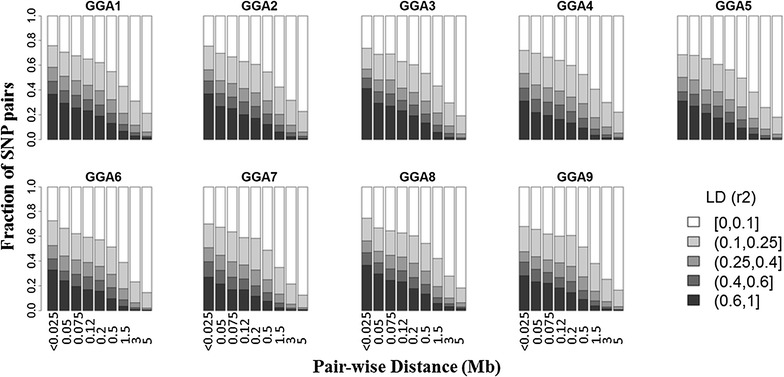
Linkage disequilibrium patterns. Level of linkage disequilibrium decay according to inter-SNP distance up to 5 Mb for the first nine chicken chromosomes (GGA1 to GGA9). The proportion of SNP pairs with different levels of linkage disequilibrium is shown for different distances between SNPs (in Mb) for the following bins (0, 0.025), (0.025, 0.05), (0.05, 0.075), (0.075, 0.12), (0.12, 0.2), (0.2, 0.5), (0.5, 1.5), (1.5, 3), (3, 5)

Results of the LD analysis are in Fig. 1 and illustrated as a plot of the LD against the physical distance of the loci up to 5 Mb. Figure 1 shows that for small distances, the level of LD was high and decreased as distance increases, especially for distances larger than 1.5 Mb. This holds true for all nine investigated chromosomes.

**Fig. 2:**
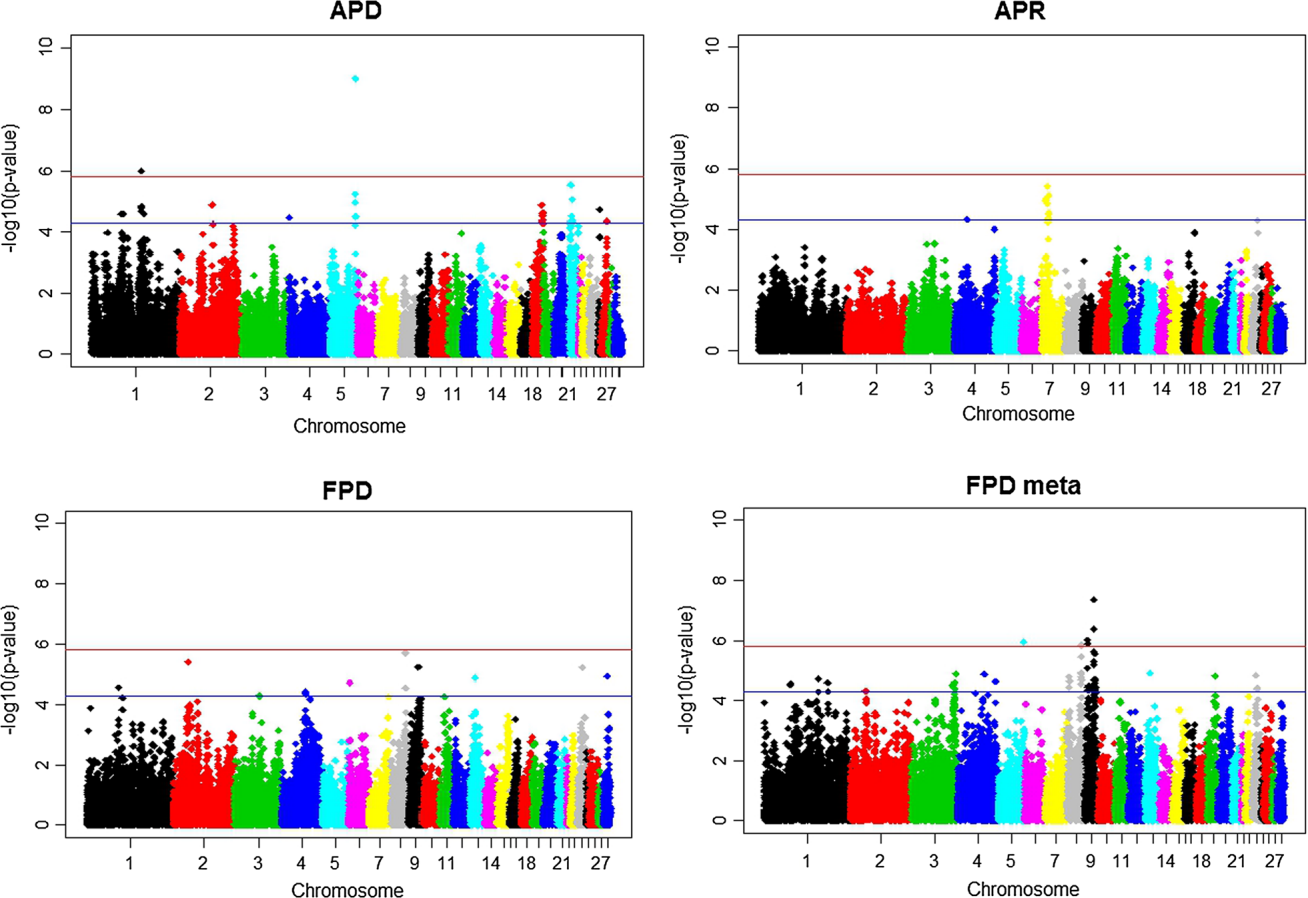
Manhattan plots. Manhattan plots of the −log_10_ *p* values for association of SNPs with APD, APR and FPD, and the meta-analysis (FDP_meta_). The *top horizontal line* indicates the genome-wide significance level $p_{genome - wide} \le 0.05$, and the *bottom line* indicates the nominal level of significance *p* ≤ 5 × 10^−5^

The GWAS (Model 1) revealed 45 (20, 19, and 58) significant SNPs at *p* < 5 × 10^−5^ for ADP (APR, FDP, and FDP_meta_). The FDR for the significant SNPs associated with ADP, APR, FDP, and FDP_meta_ were <0.025, 0.07, 0.05, and 0.01, respectively. Lists of these significant SNPs are in Additional file 1: Table S1 and Additional file 2: Table S2. Plots of the test statistics for the GWAS (i.e., −log10 *p* values) are in Fig. 2. For APD, four genome-wide significant SNPs were identified; i.e., two on GGA1 and two on GGA5 (Table 1). The latter two SNPs also showed a significant dominance effect (*p* = 0.01, results from Model 2, not shown). For FDP_meta_, nine genome-wide significant SNPs were identified (Table 1) with none showing a significant dominance effect.

**Table 1:** List of genome-wide significant SNPs for the traits APD and FDP_meta_

Trait	SNP	Chr	Position	−log10(*p*)	Gene frequency	F_ST_
APD	Gga_rs14552589	5	57353834	6.8	0.13	–
	GGaluGA290503	5	57401911	6.4	0.13	–
	Gga_rs13923655	1	116041775	6.0	0.44	–
	Gga_rs15388609	1	116062599	5.8	0.44	–
FDP_meta_	GGaluGA341482	9	17128657	7.4	0.45	0.76
	Gga_rs14676055	9	16629471	6.4	0.44	0.80
	GGaluGA341217	9	16764865	6.4	0.44	0.80
	Gga_rs13766455	9	5961337	6.0	0.46	0.82
	Gga_rs16519883	5	59368007	5.9	0.44	0.91
	Gga_rs14667686	9	6739756	5.9	0.48	0.92
	Gga_rs14652254	8	23911149	5.8	0.48	0.97
	Gga_rs15930799	8	23892743	5.8	0.48	0.97
	Gga_rs14652966	8	24679820	5.8	0.41	0.84

Chr chromosome number

Position in bp

Gene frequency in the F2-design

*P* value obtained from Model (1)

FST-value obtained from the previously conducted selection signature experiment

Results from the cluster analyses are in Tables 2 and 3. For FDP, four clusters were identified, and for FDP_meta_ 13 clusters were identified. Only the cluster on GGA8 overlapped between the two traits. Seven of the nine genome-wide significant FDP_meta_ SNPs were located within clusters on GGA8 and 9. For APD, seven clusters were identified and the four genome-wide significant SNPs were located within two clusters on GGA1 and 5. For APR, three clusters were identified on GGA7 and almost all the significant SNPs were located in clusters (see Additional file 1: Table S1 and Additional file 2: Table S2).

**Table 2:** Numbers of clusters, chromosomal positions, and numbers of significant SNPs for the traits FDP and FDP_meta_

Trait	Cluster number	Chr	Start/end position in bp 3 Mbp interval	Length in Mb	Number of SNPs *p* ≤ 5 × 10^−5^	Number of SNPs $p_{genome - wide} \le 0.05$
FPD	1	3	58,834,628–59,725,450	0.89	3	0
	2	4	53,335,653–53,945,398	0.61	6	0
	3	6	3059,760–3075 330	0.02	2	0
	4	8	25,309,634–25,399,547	0.09	2	0
FPD_meta_	1	1	58,412,953–58,831,069	0.42	3	0
	2	1	149,753,999–150,465,791	0.71	2	0
	3	2	37,372,218–39,828,657	2.46	2	0
	4	3	102,969,523–105,470,402	2.50	2	0
	5	3	107,262,448–109,945,836	2.68	3	0
	6	4	87,030,671–87,082,448	0.05	2	0
	7	8	3612,454–5410,229	1.80	3	0
	8	8	23,799,410–26,002,938	2.20	9	3
	9	9	5650,341–7645,421	2.00	5	2
	10	9	16,342,044–18,770,002	2.43	13	3
	11	9	18,726,350–20,815,056	2.09	4	0
	12	19	6883,105–8064,270	1.18	2	0
	13	24	2480,724–3900,089	1.42	3	0

Chr Chromosome

Significance level *p* ≤ 5 × 10^−5^ and *p*_*genome*-*wide*_ ≤ 0.05

**Table 3:** Numbers of clusters, chromosomal positions, and numbers of significant SNPs for the traits APD and APR

Trait	Cluster number	Chr	Start/end position in bp 3 Mbp interval	Length in Mb	Number of SNPs *p* ≤ 5 × 10^−5^	Number of SNPs $p_{genome - wide} \le 0.05$
APD	1	1	64,103,417–67,037,983	2.93	3	0
	2	1	116,041,775–117,435,846	1.39	6	2
	3	2	83,445,347–86,114,050	2.67	2	0
	4	4	33,821–552,165	0.52	7	0
	5	5	56,835,282–58,214,037	1.38	6	2
	6	18	8135,718–101,911,44	2.06	11	0
	7	21	504,778–3009,557	2.50	7	0
APR	1	7	6241,588–6327,771	0.09	3	0
	2	7	9746,560–12,631,641	2.89	10	0
	3	7	13,378,513–14,679,901	1.30	5	0

Chr Chromosome

Significance level *p* ≤ 5 × 10^−5^ and *p*_*genome*-*wide*_ ≤ 0.05

Results from the gene expression analysis are in Table 4. Nine of the 26 probe sets that showed significant results (nominal *p* value ≤0.0001) were assigned to a LOC number or were not assigned to any gene. BLAST analysis identified the corresponding gene for only one of these. The 26 probes represented 22 different genes (Table 4). Sixteen of the 1083 probes showed a significant differential expression level, among which seven had a fold difference >2, and one a fold difference of 7.8. Six of the Bonferroni’s test-corrected significant transcripts were located within the same cluster, i.e. cluster number 9. The largest number of differentially-expressed transcripts was observed on GGA9, among which eight were experiment-wide significant and four were significant probes that mapped to clusters 9 and 10.

**Table 4:** Genes located in one of the FPD_meta_ clusters (Table 2) that were significantly differentially-expressed (nominal *p* value ≤ 0.0001) in the HFP and LFP lines

ProbeSetID^a^	Chr^b^	Position (Mb)^b^	FPD_meta_ cluster	−log10 *p*	Gene symbol	Gene name	Nfold	Reg
A_87_P022983	3	104.30	4	4.58	*WDR35*	WD repeat domain 35	7.80	Up
A_87_P021624	3	104.33	4	5.85	*LAPTM4A*	lysosomal protein transmembrane 4 alpha	1.28	Down
A_87_P018137	3	104.80	4	5.23	*HS1BP3*	HCLS1 binding protein 3	2.53	Up
A_87_P254443	3	104.84	4	4.28	*LDAH*	lipid droplet associated hydrolase	1.34	Up
A_87_P176188	3	105.39	4	4.16	*LOC769627*	Unknown^c^	1.94	Down
A_87_P304288	8	3.73	7	5.64	*LOC101751271*	1-phosphatidylinositol phosphodiesterase-like	1.88	Down
A_87_P052241	8	4.03	7	4.01	*MTA1*	metastasis associated 1	1.19	Down
A_87_P079496	8	25.66	8	4.58	*GLIS1*	GLIS family zinc finger 1	2.02	Down
A_87_P016336	8	26.00	8	4.10	*TTC4*	tetratricopeptide repeat domain 4	1.37	Down
A_87_P022335	8	26.00	8	4.35	*PARS2*	prolyl-tRNA synthetase 2, mitochondrial (putative)	1.36	Up
A_87_P139413	9	5.67	9	4.17	*AQP12*	aquaporin 12	1.78	Up
A_87_P012759	9	5.67	9	6.92	*AQP12*	aquaporin 12	1.67	Up
A_87_P077026	9	5.68	9	4.09	*PAK2*	p21(RAC1)activated kinase 2	1.96	Up
A_87_P280878	9	5.69	9	7.95	*PAK2*	p21(RAC1)activated kinase 2	1.81	Up
A_87_P285338	9	5.76	9	5.38	*RNF168*	ring finger protein 168	1.27	Down
A_87_P017768	9	5.98	9	4.12	*PPP1R7*	protein phosphatase 1, regulatory (inhibitor) subunit 7	1.21	Down
A_87_P223178	9	5.98	9	4.05	*PPP1R7*	protein phosphatase 1, regulatory (inhibitor) subunit 7	1.28	Down
A_87_P023784	9	6.18	9	6.00	*ETV5*	ets variant 5	1.40	Down
A_87_P077621	9	16.69	10	4.17	*SLC12A9*	solute carrier family 12 (potassium/chloride transporters), member 9	1.51	Down
A_87_P005339	9	16.78	10	6.92	*CYP2J6L1*	cytochrome P450 2J6-like 1	2.24	Up
A_87_P177293	9	16.78	10	4.09	*CYP2J6L1*	cytochrome P450 2J6-like 1	1.97	Up
A_87_P077646	9	16.79	10	7.95	*CYP2J2L5*	cytochrome P450 2J2-like 5	2.25	Up
A_87_P181713	19	6.94	12	4.07	*FAM101B*	family with sequence similarity 101 member B	2.17	Down
A_87_P017169	19	7.26	12	4.86	*PTRH2*	peptidyl-tRNA hydrolase 2	1.16	Down
A_87_P011731	19	8.05	12	7.95	*CA4*	carbonic anhydrase IV	1.74	Down
A_87_P018194	24	25.84	13	4.19	*VPS26B*	VPS26 retromer complex component B	3.85	Up

The experiment-wide significant genes (Bonferroni corrected, *p* ≤ 0.05) are written in underline

Italic: Gene symbols

^a^Unique Agilent ID for the 60mer probe on the Agilent Chicken Gene Expression Microarrays

^b^Chromosomal assignment and position according to genome release galGal2.1

^c^Recording was discontinued and the probe set could not be assigned to any gene

**Fig. 3:**
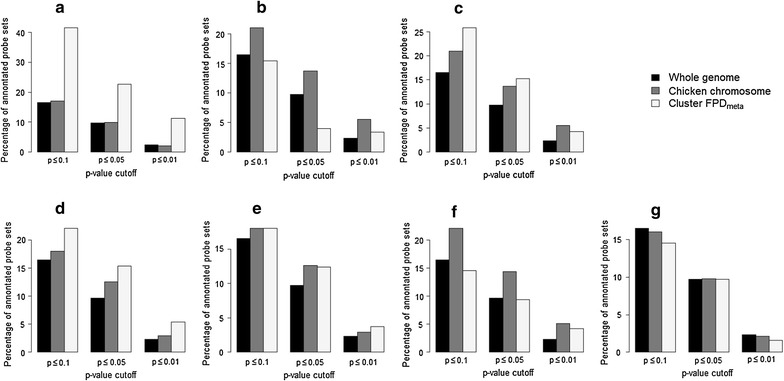
Enrichment of differentially-expressed transcripts in association clusters. **a** GGA4/Cluster FDP_meta_4, **b** GGA8/Cluster FDP_meta_7, **c** GGA8/Cluster FDP_meta_8, **d** GGA9/Cluster FDP_meta_9, **e** GGA9/Cluster FDP_meta_10, **f** GGA19/Cluster FDP_meta_12, **g** GGA24/Cluster FDP_meta_13. *Bars* depict the fractions of differentially-expressed transcripts at different *p* value thresholds at the genome- (*left bar*) and chromosome-wide (*middle bar*) level, as well as for individual clusters (*right bar*) that harbor differentially-expressed transcripts

In the previous expression study [35], 16.5, 9.7, and 2.3% of the annotated probe sets were significantly differentially-expressed with corrected *p* values <0.1, 0.05 and 0.01, respectively. For the individual chromosomes tested in this study, marked deviations from these fractions were found for GGA8 and GGA19 (Fig. 3). Among the seven FDP_meta_ clusters that harbored differentially-expressed transcripts, substantial enrichment was found for FPD_meta_ cluster 4 and a moderate enrichment for FPD_meta_ cluster 9 (Fig. 3). FDP_meta_ cluster 10 showed a slight enrichment only for *p* values <0.01 (Fig. 3).

## Discussion

### Experimental design and statistical analysis

We used an experimental F2-design, which has frequently been analyzed using classical linkage analyses. However, we applied single-marker GWAS, which was justified by the high level of LD between adjacent SNPs (Fig. 1). In addition, the decay of LD for SNPs separated by more than 1.5 Mb shows that the mapping resolution for these distances was generally high. Intuitively, this might be surprising, because it is usually assumed that an F2-design results in very long range LD. However, a recent simulation study showed that this holds true only if the founder lines of the F2 cross are ‘distantly’ related. If they are ‘closely’ related, the mapping resolution is high (and sometimes even higher than in the founder lines) [36]. In the current study, the founder lines were separated by 11 generations, and thus they can be considered to be between closely and distantly related, which resulted in the high mapping resolution for distances >1.5 Mb.

Several significant trait-associated SNPs were identified for the traits included in this study and the FDR of these significant SNPs was low. In addition, nearly all significant SNPs were located within clusters. The power to map significant FDP-associated SNPs was substantially increased by combining the results from the association mapping study in the F2 cross and the selection signature results obtained in the earlier study, as can be deduced from the roughly three-fold larger number of significant SNPs for FDP_meta_ compared to FPD. This shift in power was also observed in an experiment on bovine data [37]. Intermediate gene frequencies and high F_ST_ values (only for FDP_meta_) were obtained in the earlier selection signature experiment [20] for the genome-wide significant SNPs (see Table 1). This earlier study pointed to divergent gene frequencies in the HFP and LFP lines. Such a gene frequency pattern was expected for these genome-wide significant SNPs, because the variance contributed by an additive gene is maximized at these values. The assumption of the Fisher’s combined probability test is that the *p* values to be combined are independent. In our study, individuals from the same population were used; i.e., a sample of individuals from the HFP and LFP lines for selection signature mapping [20] and F2 individuals obtained from these lines for association mapping. However, a different type of information was used in each experiment, i.e. in the selection signature experiment differences in gene frequencies between the two lines were used, whereas in the association analysis SNP genotypes and trait phenotypes were used. A correlation of nearly 0 was found between the *p* values obtained in the selection signature and those in the association studies ($r = - 0.003$), which provided further evidence for the independence of these studies.

### Comparison of results with literature reports

Buitenhuis et al. [38] conducted a microsatellite-based linkage study to map QTL for feather pecking and identified QTL on GGA1 and 2. We also found significant clusters on these chromosomes, but a detailed comparison of the results was hampered by the wide confidence intervals obtained in the QTL linkage study. Recently, Recoquillay et al. [39] conducted a QTL linkage study for several behavior and production traits in Japanese quail. They did not detect a QTL for feather pecking but reported QTL for aggressive pecking on chromosomes 1 and 2. The corresponding position of the QTL on quail chromosome 1 on the chicken genome [39] was close to cluster number 1 for APD (Table 3), but the QTL on quail chromosome 2 could not be confirmed. Flisikowski et al. [40] suggested the genes *dopamine receptor D4* (*DRD4*) and *DEAF1 transcription factor* (*DEAF1*) as candidates for feather pecking and found significant trait associations in brain samples from the HFP and LFP lines. These lines were the same as used in Grams et al. [20] and in our study to create the F2 cross. *DRD4* and *DEAF1* are located on GGA5. We did identify one cluster for FDP_meta_ on GGA5, but it was not in the vicinity of these candidate genes. No single SNP in the chromosomal region that included these genes showed a nominal significant *p* value. In addition, although two probes were located in *DRD4* and three in *DEAF1*, none of these showed significant differential expression in the HFP and LFP lines. Thus, based on results from the current study, the candidate status of these genes was not supported.

Comparison of our study with reports from the literature revealed few congruent results, which can be due to several reasons. First, it is very likely that different ethograms were used in these studies, resulting in different definitions of the traits. Second, in addition to differences in mapping procedures and in the genetic maps used, the size of the experimental populations also differed substantially between studies, with the largest size being used in the current study. Finally, it is also possible that significant associations were not confirmed simply because they do not segregate in other populations.

### Candidate gene identification

The association clusters spanned more than 20 Mbp for all analyzed traits, i.e. a region comprising hundreds of genes, which makes the identification of candidate genes very speculative. However, inclusion of gene expression data can be used to classify positional candidate genes on a functional basis, as was done in the current study, which was based on genome-wide expression data that were restricted to association clusters to reduce the multiple-testing burden. Differentially-expressed genes that are located within QTL regions can indicate the presence of a cis-acting regulatory mutation. However, hundreds of differentially-expressed transcripts were located within the association clusters, which made such an assumption very speculative. However, enrichment of such transcripts within clusters compared to the whole genome or individual chromosomes supports the hypothesis that differential expression can, at least partly, be explained by cis-acting regulatory mechanisms. In that case, it is expected that enrichment is stronger for more stringent *p* value cutoffs. The most substantial enrichment in the current study was obtained for FDP_meta_ cluster 4 (Fig. 3). However, no functionally plausible candidate gene was identified within this region.

Positional candidate gene *SLC12A9* in FDP_meta_ cluster 10 on GGA9 exhibited experiment-wide significant differential expression between the HFP and LFP lines. However, for this cluster only a slight enrichment was observed for the most stringent *p* value cutoff. Nevertheless, *SLC12A9* remains a functionally very plausible candidate gene for this QTL. It belongs to a family of nine genes that code for electroneutral cation–chloride-cotransporters [41]. Although the function of this gene is unclear, other *SLC12* transporters are known to be crucial in the control of the electrochemical chloride gradient that is required for hyperpolarizing the postsynaptic inhibition that is mediated by GABA_A_ and glycine receptors [42]. This is remarkable, because reduction of postsynaptic GABA_A_ receptor currents is also an effect of serotonin mediated by 5-HT_2_ receptors [43]. There is a growing body of evidence that brain monoamines, such as serotonin and dopamine, are involved in the occurrence of feather pecking and aggressive pecking in hens [44–48] and in aggressive behavior in humans [49]. Kops et al. [47] showed that differences in dopamine turnover between a low mortality and a control hen line were largest, in particular, in the arcopallium region of the brain. Another purely positional candidate gene for feather pecking was located in FDP_meta_ cluster 9, i.e. *CLSTN2* (*calsyntenin 2*), which is also involved in postsynaptic signaling related to excitatory synaptic transmission [50].

For APD, the *GNG2* (*G protein subunit gamma 2*) gene was identified as a positional candidate gene in FDP_meta_ cluster 5 on GGA5 (Table 3). This gene is also involved in monoamine signaling, particularly in postsynaptic signaling at serotonergic (KEGG pathway ko04726) and dopaminergic (KEGG pathway ko04728) synapses.

### Shared environment and associated effects

Behavior traits involve interactions between individuals. Statistical models that include interaction or associated effects have developed, as reviewed by Bijma [51] and Ellen et al. [52], which have shown that these effects can substantially contribute to the heritable variation in survival of hens related to feather pecking and cannibalism [52]. Indeed, these interactions might also be another possible explanation for the low genetic trend in later generations in our selection experiment [20]. In a recent study, we chose the simplest form to capture shared environment effects and associated effects by fitting a random pen effect to the model [17]. Since pen variances were very small, they were not included in the current study. Moreover, the size of the pens used here was rather large for social interaction models.

## Conclusions

Several significant trait-associated clusters of SNPs were identified, especially for the trait FPD_meta_ but also for aggressive pecking. However, behavioral traits, appeared to be controlled by many genes with small effects and no single SNP was promising for selection purposes. However, understanding the motivation for feather pecking is of interest in its own right. In-depth sequence-based association analyses of the clusters identified in this study and subsequent identification of candidate genes from a small list of putative positional genes will help to formulate and validate hypotheses for the expression of this abnormal behavior pattern. Clearly, for this purpose additional data need to be collected.

## Additional files

**Additional file 1: Table S1.** List of significant SNPs with *p* ≤ 5 × 10^−5^, their chromosomal regions and their *p* values for the trait feather pecks delivered (FPD) and the meta-analysis (FPD_meta_).

**Additional file 1: Table S2.** List of significant SNPs with *p* ≤ 5 × 10^- 5^, their chromosomal regions and their *p* values for traits aggressive pecks delivered (APD) and aggressive pecks received (APR).
