# Supplementary material for: A genome-wide association study in a large F2-cross of laying hens reveals novel genomic regions associated with feather pecking and aggressive pecking behavior
Source: Genet Sel Evol. 2017 Feb 3;49:18. doi: 10.1186/s12711-017-0287-4 (PMC5291977; doi:10.1186/s12711-017-0287-4)
Supplement: Supplementary file 1 — Additional file 1: Table S1. List of significant SNPs with p ≤ 5 × 10^−5^, their chromosomal regions and their p values for the trait feather pecks delivered (FPD) and the meta-analysis (FPDmeta). [file 12711_2017_287_MOESM1_ESM.docx]

**Table S1** List of significant SNPs with a p-value ≤ 5x10^-5^, their chromosomal region and their p-value for trait feather pecks delivered (FPD) and the Metal-Analysis (FPD_meta_).

| **Trait** | **SNP** | **Chr** | **Position** | **-log10(p)** | **Cluster** |
| --- | --- | --- | --- | --- | --- |
| FPD | GGaluGA023840 | 1 | 69812429 | 4.569 | - |
|  | Gga_rs13669267 | 2 | 37372218 | 5.398 | - |
|  | GGaluGA224037 | 3 | 58834628 | 4.319 | 1 |
|  | GGaluGA224156 | 3 | 59186202 | 4.319 | 1 |
|  | GGaluGA224309 | 3 | 59725450 | 4.319 | 1 |
|  | Gga_rs13521814 | 4 | 53375709 | 4.444 | 2 |
|  | Gga_rs14469602 | 4 | 53397816 | 4.444 | 2 |
|  | Gga_rs14469696 | 4 | 53453267 | 4.420 | 2 |
|  | Gga_rs14469702 | 4 | 53461607 | 4.444 | 2 |
|  | Gga_rs15579522 | 4 | 53892204 | 4.357 | 2 |
|  | Gga_rs14470197 | 4 | 53945398 | 4.357 | 2 |
|  | Gga_rs16526327 | 6 | 3059760 | 4.721 | 3 |
|  | Gga_rs13561199 | 6 | 3075330 | 4.721 | 3 |
|  | Gga_rs14653727 | 8 | 25309634 | 4.553 | 4 |
|  | GGaluGA331049 | 8 | 25399547 | 5.699 | 4 |
|  | GGaluGA341482 | 9 | 17128657 | 5.222 | - |
|  | GGaluGA093070 | 13 | 7103987 | 4.886 | - |
|  | Gga_rs13605122 | 24 | 3315257 | 5.222 | - |
|  | Gga_rs13546091 | 28 | 2140227 | 4.921 | - |
| FPD_meta_ | GGaluGA019519 | 1 | 58412953 | 4.548 | 1 |
|  | GGaluGA019545 | 1 | 58477922 | 4.516 | 1 |
|  | Gga_rs14828914 | 1 | 58537760 | 4.543 | 1 |
|  | GGaluGA042442 | 1 | 127308590 | 4.727 | - |
|  | Gga_rs13712580 | 1 | 149753999 | 4.590 | 2 |
|  | Gga_rs13712669 | 1 | 150001928 | 4.300 | 2 |
|  | Gga_rs13669267 | 2 | 37372218 | 4.327 | 3 |
|  | GGaluGA142420 | 2 | 39486006 | 4.325 | 3 |
|  | Gga_rs13702304 | 3 | 103609224 | 4.511 | 4 |
|  | Gga_rs13702581 | 3 | 104349320 | 4.437 | 4 |
|  | Gga_rs14408039 | 3 | 107262448 | 4.388 | 5 |
|  | GGaluGA238045 | 3 | 107786255 | 4.601 | 5 |
|  | Gga_rs13525874 | 3 | 109945836 | 4.890 | 5 |
|  | Gga_rs16419024 | 4 | 59770870 | 4.886 | - |
|  | Gga_rs14500076 | 4 | 87030671 | 4.650 | 6 |
|  | Gga_rs15637216 | 4 | 87076909 | 4.627 | 6 |
|  | **Gga_rs16519883** | 5 | 59368007 | 5.948 | - |
|  | Gga_rs15900019 | 8 | 4002499 | 4.434 | 7 |
|  | Gga_rs10723790 | 8 | 4211591 | 4.788 | 7 |
|  | GGaluGA323765 | 8 | 5410229 | 4.641 | 7 |
|  | **Gga_rs15930799** | 8 | 23892743 | 5.844 | 8 |
|  | **Gga_rs14652254** | 8 | 23911149 | 5.844 | 8 |
|  | Gga_rs14652925 | 8 | 24646720 | 4.544 | 8 |
|  | **Gga_rs14652966** | 8 | 24679820 | 5.839 | 8 |
|  | GGaluGA330724 | 8 | 24758580 | 5.460 | 8 |
|  | Gga_rs15932003 | 8 | 24834266 | 4.928 | 8 |
|  | GGaluGA330792 | 8 | 24921219 | 4.783 | 8 |
|  | GGaluGA330826 | 8 | 24980300 | 4.888 | 8 |
|  | GGaluGA331049 | 8 | 25399547 | 4.706 | 8 |
|  | Gga_rs15962845 | 9 | 5754325 | 5.069 | 9 |
|  | **Gga_rs13766455** | 9 | 5961337 | 6.017 | 9 |
|  | **Gga_rs14667686** | 9 | 6739756 | 5.891 | 9 |
|  | Gga_rs14667611 | 9 | 6812627 | 4.339 | 9 |
|  | GGaluGA336891 | 9 | 7219744 | 4.453 | 9 |
|  | Gga_rs14665188 | 9 | 9007799 | 4.502 | - |
|  | Gga_rs13608664 | 9 | 16342044 | 4.388 | 10 |
|  | Gga_rs14675951 | 9 | 16548816 | 5.197 | 10 |
|  | Gga_rs15976485 | 9 | 16608755 | 5.307 | 10 |
|  | **Gga_rs14676055** | 9 | 16629471 | 6.392 | 10 |
|  | GGaluGA341173 | 9 | 16688339 | 5.287 | 10 |
|  | **GGaluGA341217** | 9 | 16764865 | 6.376 | 10 |
|  | GGaluGA341245 | 9 | 16810500 | 5.625 | 10 |
|  | GGaluGA341277 | 9 | 16849902 | 4.677 | 10 |
|  | GGaluGA341293 | 9 | 16877654 | 4.536 | 10 |
|  | **GGaluGA341482** | 9 | 17128657 | 7.368 | 10 |
|  | GGaluGA341680 | 9 | 17579192 | 4.706 | 10 |
|  | Gga_rs14677433 | 9 | 18297108 | 4.567 | 10 |
|  | Gga_rs14677551 | 9 | 18425812 | 5.558 | 10 |
|  | Gga_rs15979368 | 9 | 19293924 | 4.692 | 11 |
|  | Gga_rs16006794 | 9 | 19411600 | 4.382 | 11 |
|  | Gga_rs16742615 | 9 | 19427105 | 4.361 | 11 |
|  | GGaluGA342417 | 9 | 20815056 | 4.426 | 11 |
|  | GGaluGA093070 | 13 | 7103987 | 4.924 | - |
|  | Gga_rs14122190 | 19 | 6883105 | 4.824 | 12 |
|  | GGaluGA127801 | 19 | 6896487 | 4.819 | 12 |
|  | GGaluGA191629 | 24 | 2533040 | 4.840 | 13 |
|  | Gga_rs13605122 | 24 | 3315257 | 4.376 | 13 |
|  | Gga_rs14295311 | 24 | 3419984 | 4.418 | 13 |

The genome-wide significant SNPs (Bonferroni corrected, p ≤ 0.05) are written in boldface
