# Supplementary material for: A genome-wide association study in a large F2-cross of laying hens reveals novel genomic regions associated with feather pecking and aggressive pecking behavior
Source: Genet Sel Evol. 2017 Feb 3;49:18. doi: 10.1186/s12711-017-0287-4 (PMC5291977; doi:10.1186/s12711-017-0287-4)
Supplement: Supplementary file 2 — Additional file 1: Table S2. List of significant SNPs with p ≤ 5 × 10- 5, their chromosomal regions and their p values for traits aggressive pecks delivered (APD) and aggressive pecks received (APR). [file 12711_2017_287_MOESM2_ESM.docx]

**Table S2** List of significant SNPs with a p-value ≤ 5x10^-5^, their chromosomal region and their p-value for trait aggressive pecks delivered (APD) and aggressive pecks received (APR).

| **Trait** | **SNP** | **Chr** | **Position** | **-log10(p)** | **Cluster** |
| --- | --- | --- | --- | --- | --- |
| APD | Gga_rs14834942 | 1 | 64103417 | 4.548 | 1 |
|  | Gga_rs14837208 | 1 | 66726295 | 4.550 | 1 |
|  | Gga_rs15296857 | 1 | 67037983 | 4.561 | 1 |
|  | **Gga_rs13923655** | 1 | 116041775 | 6.024 | 2 |
|  | **Gga_rs15388609** | 1 | 116062599 | 5.838 | 2 |
|  | Gga_rs15388671 | 1 | 116098957 | 4.797 | 2 |
|  | Gga_rs13923933 | 1 | 116353270 | 4.632 | 2 |
|  | Gga_rs14875693 | 1 | 116457611 | 4.748 | 2 |
|  | GGaluGA038883 | 1 | 116538528 | 4.835 | 2 |
|  | Gga_rs16738272 | 1 | 121495752 | 4.568 | - |
|  | Gga_rs14210222 | 2 | 83445347 | 4.841 | 3 |
|  | Gga_rs13636444 | 2 | 86114050 | 4.886 | 3 |
|  | Gga_rs14418954 | 4 | 33821 | 4.456 | 4 |
|  | Gga_rs14418847 | 4 | 310336 | 4.456 | 4 |
|  | Gga_rs14418830 | 4 | 332468 | 4.456 | 4 |
|  | Gga_rs14418813 | 4 | 360213 | 4.456 | 4 |
|  | Gga_rs14418723 | 4 | 518571 | 4.456 | 4 |
|  | Gga_rs14418713 | 4 | 540435 | 4.456 | 4 |
|  | Gga_rs14418710 | 4 | 552165 | 4.456 | 4 |
|  | Gga_rs14552049 | 5 | 56835282 | 4.465 | 5 |
|  | Gga_rs14552339 | 5 | 57143305 | 5.259 | 5 |
|  | Gga_rs14552504 | 5 | 57246827 | 4.952 | 5 |
|  | **Gga_rs14552589** | 5 | 57353834 | 6.829 | 5 |
|  | **GGaluGA290503** | 5 | 57401911 | 6.400 | 5 |
|  | GGaluGA290917 | 5 | 58214037 | 4.485 | 5 |
|  | Gga_rs15832113 | 18 | 8658969 | 4.854 | 6 |
|  | GGaluGA122356 | 18 | 8928217 | 4.324 | 6 |
|  | GGaluGA122973 | 18 | 9699487 | 4.353 | 6 |
|  | Gga_rs15469578 | 18 | 9898566 | 4.590 | 6 |
|  | GGaluGA123114 | 18 | 9906133 | 4.512 | 6 |
|  | GGaluGA123129 | 18 | 9928246 | 4.514 | 6 |
|  | GGaluGA123133 | 18 | 9932567 | 4.514 | 6 |
|  | Gga_rs16347507 | 18 | 9945623 | 4.514 | 6 |
|  | Gga_rs14417028 | 18 | 9949736 | 4.514 | 6 |
|  | Gga_rs16347539 | 18 | 9983890 | 4.599 | 6 |
|  | Gga_rs16347624 | 18 | 10029434 | 4.599 | 6 |
|  | Gga_rs16177511 | 21 | 1095000 | 4.320 | 7 |
|  | Gga_rs16177666 | 21 | 1199251 | 5.475 | 7 |
|  | GGaluGA182481 | 21 | 1204493 | 5.475 | 7 |
|  | GGaluGA182516 | 21 | 1266391 | 5.501 | 7 |
|  | GGaluGA182786 | 21 | 1914059 | 4.494 | 7 |
|  | GGaluGA183181 | 21 | 2414911 | 5.067 | 7 |
|  | GGaluGA183305 | 21 | 2583575 | 4.349 | 7 |
|  | Gga_rs16072064 | 25 | 1438046 | 4.719 | - |
|  | Gga_rs16203090 | 26 | 3510820 | 4.338 | - |
| APR | Gga_rs14443929 | 4 | 30737168 | 4.310 | - |
|  | GGaluGA310577 | 7 | 6241588 | 4.959 | 1 |
|  | Gga_rs14604136 | 7 | 6325307 | 4.959 | 1 |
|  | Gga_rs14604144 | 7 | 6327771 | 4.959 | 1 |
|  | Gga_rs15838552 | 7 | 9746560 | 4.959 | 2 |
|  | Gga_rs14605439 | 7 | 10476794 | 4.959 | 2 |
|  | Gga_rs15839283 | 7 | 10505869 | 4.959 | 2 |
|  | GGaluGA311212 | 7 | 10510834 | 4.959 | 2 |
|  | Gga_rs15839450 | 7 | 10582674 | 5.000 | 2 |
|  | Gga_rs15839686 | 7 | 10726089 | 5.000 | 2 |
|  | Gga_rs15840596 | 7 | 11199463 | 5.000 | 2 |
|  | Gga_rs13742807 | 7 | 12394545 | 5.398 | 2 |
|  | Gga_rs10729685 | 7 | 12514023 | 4.824 | 2 |
|  | GGaluGA311997 | 7 | 12631641 | 4.824 | 2 |
|  | Gga_rs14608583 | 7 | 13420198 | 4.347 | 3 |
|  | GGaluGA312585 | 7 | 13933219 | 4.367 | 3 |
|  | GGaluGA312831 | 7 | 14595204 | 4.509 | 3 |
|  | Gga_rs15846766 | 7 | 14652969 | 5.097 | 3 |
|  | GGaluGA312856 | 7 | 14679901 | 4.509 | 3 |

The genome-wide significant SNPs (Bonferroni corrected, p ≤ 0.05) are written in boldface.
